# Dietary Intakes and Supplement Use in Pre-Adolescent and Adolescent Canadian Athletes

**DOI:** 10.3390/nu8090526

**Published:** 2016-08-26

**Authors:** Jill A. Parnell, Kristin P. Wiens, Kelly A. Erdman

**Affiliations:** 1 Department of Health and Physical Education, Mount Royal University, 4825 Mount Royal Gate SW, Calgary, AB T3E 6K6, Canada; 2 Department of Behavioral Health and Nutrition, University of Delaware, 026 North College Avenue, Newark, DE 19716, USA; kwiens@udel.edu; 3 Sport Medicine Centre, University of Calgary, 2500 University Dr NW, Calgary, AB T2N 1N4, Canada; kerdman@csicalgary.ca

**Keywords:** diet analysis, youth athletes, nutrient intakes, dietary supplements, ergogenic aids

## Abstract

Young athletes experience numerous dietary challenges including growth, training/competition, unhealthy food environments, and travel. The objective was to determine nutrient intakes and supplement use in pre-adolescent and adolescent Canadian athletes. Athletes (*n* = 187) aged 11–18 years completed an on-line 24-h food recall and dietary supplement questionnaire. Median energy intake (interquartile range) varied from 2159 kcal/day (1717–2437) in 11–13 years old females to 2905 kcal/day (2291–3483) in 14–18 years old males. Carbohydrate and protein intakes were 8.1 (6.1–10.5); 2.4 (1.6–3.4) in males 11–13 years, 5.7 (4.5–7.9); 2.0 (1.4–2.6) in females 11–13 years, 5.3 (4.3–7.4); 2.0 (1.5–2.4) in males 14–18 y and 4.9 (4.4–6.2); 1.7 (1.3–2.0) in females 14–18 years g/kg of body weight respectively. Median vitamin D intakes were below the recommended dietary allowance (RDA) and potassium was below the adequate intake (AI) for all athlete groups. Females 14–18 years had intakes below the RDA for iron 91% (72–112), folate 89% (61–114) and calcium 84% (48–106). Multivitamin-multiminerals, vitamin C, vitamin D, vitamin-enriched water, protein powder, sport foods, fatty acids, probiotics, and plant extracts were popular supplements. Canadian pre-adolescent and adolescent athletes could improve their dietary intakes by focusing on food sources of calcium, vitamin D, potassium, iron, and folate. With the exceptions of vitamin D and carbohydrates during long exercise sessions, supplementation is generally unnecessary.

## 1. Introduction

Young athletes experience numerous nutritional challenges including: Meeting nutrient needs for growth, training/competition, the maintenance of health, concurrent sporting pursuits, challenging schedules (school, training, socializing, work, etc.), a lack of knowledge, a reliance on others for the purchase and preparation of foods, unhealthy eating environments in their locations of training and competition, and travel [1,2,3,4]. Additionally, the eating patterns and attitudes towards foods, set during adolescence, can impact an individual’s lifelong relationship with food and nutrition [1]. Currently, targeted recommendations for youth are lacking, forcing the default use of adult recommendations.

Nutrient needs during adolescence are relatively high as compared to adulthood to support development [5] and these athletes require reliable guidance to ensure health and the prevention of injuries as they progress in their sporting endeavors. Dietary intakes in young athletes have been found to be superior to their non-athletic counterparts [6,7,8]. Conversely, the increased demands of intense physical activity imply that the consequences of a deficiency are greater in this demographic. Furthermore, young athletes may have increased nutrient needs suggesting a direct comparison to intakes in non-athletes does not accurately represent the physiological impact of their dietary intakes. In young athletes, nutrients identified as of concern due to insufficient intakes include: Carbohydrates (especially during exercise), vitamin E, vitamin D, calcium, iron, magnesium, and zinc [5,9,10,11]. There is also a concern that the pressures associated with athletic performance can promote eating disorders with increased rates typically found in adult elite athletes as compared to non-athletes [12]. The role of sport in fostering eating disorders in youth is controversial with some finding a protective effect in young, female athletes as compared to non-athletes [13]. Additional investigation is required though it appears that the level of competition and sport type are critical with an increased risk in elite athletes as compared to recreational level athletes and those competing in sports that encourage leanness [13,14].

Few studies have evaluated the dietary patterns of young Canadian athletes, particularly those participating in sport at the community/provincial level. The primary aim of this study was to quantify dietary intakes in young Canadian athletes, from a wide variety of sports, competing primarily at the community or provincial level. A secondary aim was to evaluate dietary supplement use in the contexts of nutrient needs, health, and performance.

## 2. Materials and Methods

### 2.1. Participants

Male (*n* = 84) and female (*n* = 103) athletes aged 11 to 18 years were recruited from the province of Alberta through sporting communities and the public school system. The term “athlete” was defined as competing at the city level or higher and training ≥5 h per week. A sample size of 150 allowed the reporting of estimates to a margin of error of no more than 8% with a 95% confidence level [15].

### 2.2. Procedures

Athletes and/or their parents/guardians completed the Food Behaviour Questionnaire (FBQ). The FBQ is a web-based 24-h diet recall developed by Canadian researchers for diet analysis in youth [16]. Sport drinks were included in the questionnaire; however, other sport foods and supplements were not included, as they could not be linked to the Canadian Nutrient File for analysis. Questions regarding meal timing and training/competition were added to the FBQ. Basal metabolic rate (BMR) was calculated by the Schofield equation and participants with energy intake/BMR ≤0.89 were classified as low energy reporters [17] and removed from analyses.

A dietary supplement questionnaire [18] was incorporated into the FBQ and included sport foods, vitamin/mineral supplements, fatty acid supplements, plant extracts, probiotics, and ergogenic aids. All questionnaires were previously tested for reliability and validity [16,18]; however, the dietary supplement questionnaire was originally delivered in a face-to-face manner. Consequently, the electronic format was tested for reliability in a subset of 21 athletes who completed both a paper and electronic version. Weighted kappa coefficients [19] found questions had fair to moderate agreement with the exception of frequency of use for vitamin E, vitamin-enriched water, glutamine, sport drinks, recovery drinks, probiotics, sport gels/gummies, and caffeine pills.

### 2.3. Ethical Considerations

Individualized access information for the consent form and questionnaire was sent to the parent’s/guardian’s email account (except for those 18 years of age). Parental consent and athlete assent was provided electronically prior to commencing the questionnaire. The Mount Royal University Human Research Ethics Board (2013-19) and a school board (2013 1127 L) approved the study.

### 2.4. Statistical Analyses

Athletes were categorized based on Dietary Reference Intakes (DRIs): Male 11–13 years, female 11–13 years, male 14–18 years, and female 14–18 years. Estimated energy requirements (EER) were determined using the Institute of Medicine’s EER equations and an “active” value for physical activity [20] (pp. 181–182). For macronutrient intakes, absolute values, intakes adjusted for body weight, and percent of total energy intake were calculated. Micronutrients are presented as absolute values and the percent of the Recommended Dietary Allowance (RDA) or Adequate Intake (AI). Percent RDA or AI was calculated by taking the intake for each athlete and dividing it by the established RDA or AI for that age and gender [21] and multiplying by 100. Supplement use was quantified as the percent of respondents who consumed the supplement Regularly, Occasionally (“Specific Times” or “I’ve Tried It”), and Never (“Never” or “Unfamiliar”). Data was checked for normality using the Shapiro-Wilk test. Differences for macronutrients in the percent of total calories and g/kg/body weight and micronutrient percent of Recommended Dietary Allowance (%RDA) or Adequate Intake (%AI) [21] were determined using a Kruskal–Wallis non-parametric test and difference between genders and age groups were assessed using Dunn’s test for post-hoc pairwise comparisons. In cases where the data was normally distributed, a one-way ANOVA with a Bonferroni post-hoc comparison within age and gender was also calculated. Differences in percent of athletes meeting Canada’s Food Guide [22] servings and dietary supplement/ergogenic aids use were determined using a Pearson’s Chi-squared test and the effect size reported as V = Cramer’s V. A *p*-value of < 0.05 was considered to be statistically significant. All analyses were performed using SPSS version 22 (IBM Corporation, Armonk, NY, USA) and Stata 14 (StataCorp, College Station, TX, USA).

## 3. Results

### 3.1. Participant Characteristics

The FBQ was completed by 187 participants, however, two females 11–13 years, two males 11–13 years, 12 females 14–18 years, and three males 14–18 years were identified as low-energy reporters and were removed from the analyses. Descriptive characteristics for the remaining 168 participants who completed the FBQ are presented in Table 1. Eight percent of young females, 15% of older females, 4% of young males, and 0% of older males reported trying to lose weight (*p* = 0.030; V = 0.204), whereas 3% of young females, 0% of older females, 15% of young males, and 42% of older males reported trying to gain weight (*p* < 0.001; V = 0.381). Energy intakes as a percent of EER were 98% (83–125) in 11–13 years old males, 91% (76–112) in 11–13 years old females, 85% (70–107) in 14–18 years old males, and 89% (73–104) in 14–18 years old females; there were no statistically significant differences between the groups.

### 3.2. Nutrient Intakes

Macronutrient intakes are presented in Table 2. Younger males had greater carbohydrate intakes based on body weight as compared to older males (*p* = 0.001) and older females (*p* < 0.001). Protein intakes based on body weight were highest in young males and greater as compared to older females (*p* < 0.001) and older males (*p* = 0.046), however the dietary assessment did not include protein supplements. The percent of calories coming from sugar and fats was consistent across all groups, however, based on body weight, young males had greater intakes of total fat as compared to older females (*p* < 0.001).

Micronutrient intakes are described in Table 3. Athletes’ median intakes met or exceeded the RDA for all vitamins with the exception of vitamin D, folate (14–18 years females) and vitamin A (females 11–13 years and males 14–18 years). Females did not consume the RDA for calcium and older females did not meet the RDA for iron. Athletes did not meet the AI for potassium. Sodium intakes exceeded the upper limit in 89% of males 11–13 years, 65% of females 11–13 years, 83% of males 14–18 years, and 69% of females 14–18 years (*p* = 0.061; V = 0.209).

Servings according to Eating Well with Canada’s Food Guide can be found in Table 4. Differences between the groups were noted in milk and alternatives where older females had the fewest percent of participants meeting the recommendation (*p* = 0.010; V = 0.259) and in the meat and alternatives where older males had a lower percent meeting the recommendations (*p* = 0.002; V = 0.294).

### 3.3. Supplements

The use of dietary supplements and ergogenic aids is presented as those who consume the supplement regularly, occasionally, or never (Table 5). Given the broad definition of dietary supplements and the inclusion of occasional use over the past 3 months, 100% of athletes reported some usage. Multivitamin-multiminerals, vitamin C, vitamin D, sport bars, and protein powders were the most commonly consumed supplements on a regular basis. Supplements athletes frequently reported using occasionally included vitamin C, vitamin-enriched water, protein powders, sport bars and drinks, plant extracts, and gels/gummies. When supplement use was analyzed according to gender alone, differences were noted in fatty acid intakes with 17% of males reporting regular use, 5% occasional use and 78% never, whereas females reported regular use at 8%, occasional use at 15% and never at 77% (*p* = 0.045; V = 0.189). Furthermore, 18% of males used sport drinks regularly, 69% occasionally and 13% never as compared to females at 7% regularly, 70% occasionally, and 23% never (*p* = 0.040; V = 0.193). Analysis by age groups 11–13 years and 14–18 years found increased use of protein powder in the older age group with regular use 18%, occasional 45%, and never 37% vs. 10% regular, 23% occasional, and 68% never in 11–13 years (*p* = 0.001; V = 0.296). None of the athletes reported regular use of energy drinks, however, 14–18 years old had occasional use at 27% vs. 3% in 11–13 years (*p* < 0.001; V = 0.294). There were no other significant differences between gender or age groups in supplement use.

## 4. Discussion

This research is significant as it analyzes dietary intakes and supplement use across a broad range of sport types in a cohort of younger, understudied athletes. Studies focused on a single sport are extremely valuable to the cohort of athletes they represent, however, are less able to provide information regarding general nutrition concerns.

### 4.1. Overall Diet Quality and Energy Intakes

Adolescents typically adhere to a dietary pattern that is energy dense, however, low in nutrients [23]. Conversely, studies do report improved dietary intakes in adolescent athletic populations as compared to their sedentary peers [6,7,8]. Athletes have increased rates of disordered eating as compared to non-athletes, however, this concerns is more applicable to older athletes competing at higher levels than the majority of our sample [12]. Notably, the decline in diet quality in our female 14–18 years cohort, as compared to other groups, could be indicative of a trend towards increased rates of disordered eating; coaches and parents should carefully monitor this group. When athletes self-reported on the quality of their diet, 3% thought their diet was poor, 72% rated their diet as average, and 25% selected excellent.

Assessment of the athletes’ Canada’s Food Guide servings can provide an understanding of overall diet quality, however, Canada’s Food Guide does not take into consideration high levels of physical activity. Consequently, it is possible that Table 4 underestimates the percentage of athletes consuming insufficient numbers of servings. It can reasonably be concluded that milk and alternatives is a food category of concern for young female athletes, a risk that increases with age. Furthermore, all athletes need to be encouraged to consume more vegetables and fruits. Another factor, potentially limiting performance in some athletes, may be inadequate intakes of whole grains, as grains are often good sources of carbohydrates, food folic acid, and iron. The relatively high contribution of sugars and saturated fats to total calories and servings from the “other” category, suggest young athletes need to focus on healthier choices and reducing processed and fast foods. A conclusion further supported by the high sodium intakes.

Energy intakes are slightly lower than the EER. An energy deficit is commonly reported in athletes [5,24,25], however, it is extremely difficult to accurately assess energy needs in young athletes [1] and self-reported dietary records are limited. Furthermore, BMI data indicates that underweight is not a prominent concern in this cohort of athletes.

### 4.2. Macronutrients

It is difficult to assess the adequacy of macronutrient intakes in young athletes due to a lack of recommendations and a default to adult values [26,27]. Carbohydrate recommendations based on body weight rather than percent of total calories are thought to best reflect athlete needs and range from 3 to 12 g/kg/BW depending on exercise load [28]. Carbohydrate intakes meet the minimum requirements in all our athlete groups. Importantly, when carbohydrate intakes were analyzed by sport type, endurance and intermittent athletes had median intakes of 5.5 g/kg/BW; whereas, aesthetic and power athletes had higher intakes at 6.2 g/kg/BW and 5.8 g/kg/BW respectively. Therefore, it may be advisable to emphasize carbohydrates for young endurance athletes. Inadequate carbohydrate intake in young athletes has been noted in other studies [5,11,26,27]. Dietary fibre intakes did not meet recommendations, suggesting that information on the quantity and quality of carbohydrates should be provided.

Protein recommendations have recently increased and range from 1.2 to 2.0 g/kg/BW depending on the type and amount of physical activity [29]. Furthermore, it has been estimated that protein intakes of 1.5 g/kg BW are required for nitrogen balance in young sprinters [30] and can, therefore, be assumed sufficient for most young athletes. Average protein intakes, from food alone, exceeded the upper recommendation for performance in all groups except 14–18 years old females, suggesting that this is not a nutrient of concern for young Canadian athletes; a finding supported by others [26,27]. Data in young, elite Canadian soccer players reports average protein intakes of 1.8 g/kg/BW [5]. Indeed, even elite, young female figure skaters reported an average protein intake of 1.2 g/kg/BW, despite total caloric intakes 44% less than their estimated needs [25]. It has been suggested that protein intakes are generally adequate or excessive because protein is overvalued among coaches and athletes [9,31].

Dietary fats ranged from 31% to 33% of total calories, which is at the high-end of the recommended 25%–35% [20]. Intakes of at least 30% have been consistently reported in adolescent athletes [1]. Young athletes may benefit from consuming fewer calories from fat and increasing calories from carbohydrates [11]. Conversely, if a young athlete were struggling to meet their caloric needs, healthy fats would be the most energy dense options.

### 4.3. Micronutrients

Athletes may have a greater need for select micronutrients, however, no widely accepted values are available [29]. Athletes met or exceeded the micronutrient recommendations in most cases. Notably, however, females between 14 and 18 years tended to have the lowest intakes relative to other groups, highlighting this group as higher risk. Importantly, when considering the micronutrient values as determined from 24-h recalls there is the possibility of underreporting. Underreporting was addressed in the methods by identifying low energy reporters, however could still be present to a lesser degree in the remaining sample, possibly inflating nutrient deficiencies.

Of particular apprehension in young athletes, especially females, is their ability to meet the recommendations for the bone-building nutrients such as vitamin D, calcium, and phosphorus [25]. Low bone mineral density has been reported in adolescent, female athletes, primarily those with amenorrhea [32]. Our results indicate vitamin D and calcium are of concern, with a high percentage of female athletes not meeting the RDA for these nutrients. Gibson et al. [5] also highlight vitamin D and calcium as nutrients of concern for young Canadian soccer players. The inadequate intakes of bone-related nutrients in our population may be associated with lower intakes of milk and alternatives.

Iron and folate (females 14–18 years) were also identified as nutrients where intakes often did not meet the RDA. Deficiencies in either of these nutrients can promote anemia, which can cause fatigue and suboptimal athletic performance [33]. Young female athletes are at a higher risk than their male counterparts for iron deficiencies due to losses from menses and lower energy intakes, and compared to their sedentary peers due to the increased requirements for intense training [29,33,34]. Diet assessments in other athletic populations also find inadequate intakes for iron and/or folate in young female athletes [5,10,11,25]. In Gibson et al. [5] although only 6% of athletes had iron intakes below the EAR, 89% had serum ferritin levels below 35 μg/L, a level thought to negatively impact performance.

Potassium was another nutrient where athletes did not meet the AI. Potassium has a role in muscle and nerve function and, consequently, is related to athletic performance. Although it is questionable whether intakes in the range noted here could negatively impact performance, it would still be prudent for athletes to increase their vegetable and fruit intakes to meet the Canada’s Food Guide recommendations, as these foods are often good sources of potassium.

### 4.4. Supplements

Sport nutrition professionals agree that athletes should not use dietary supplements to compensate for a poor diet but rather consume whole foods to meet their nutrient needs. Only when this is not feasible should athletes rely on supplements [1,33].

Protein supplements were popular in our cohort; yet appear to be unnecessary given the high dietary intakes. Additionally, the need for a multivitamin-multimineral, vitamin C, and vitamin-enriched waters is questionable in the context of their nutrient intakes from food alone. Vitamin D supplementation is often recommended for athletes living at northern latitudes or training indoors and levels should be carefully monitored in young Canadian athletes to determine if supplementation is necessary [29]. Although calcium has been identified as a nutrient of concern in young female athletes, dietary changes rather than supplementation is recommended. Sport drinks, gummies, and gels could be justified during longer training sessions, as insufficient carbohydrate during exercise has been identified as a weakness in some young athletes [27]. Athletes should be cautious, however, to use these products only as needed and monitor calorie and sugar intakes [35]. Probiotics have the potential to reduce gastrointestinal symptoms and respiratory illnesses in athletes, however, additional research is needed to confirm these benefits and determine the types and effective dose [36].

Protein supplementation and energy drink consumption increased with age, a trend that we have previously reported [18]. Furthermore, others have found that dietary supplement use in youth increases with age [37]. Additional research is required to determine the reasons for these trends. However, it should be noted that the older athletes were also more likely to compete at a higher level; thus, the level of competition could be a factor, as these products are often viewed as performance-enhancing. Regardless, energy drink consumption is concerning in this demographic as they are contraindicated for those under the age of 18 years [35]. Male athletes were also more likely to use sport drinks. The reason for this is unclear, however, it may be linked to an overall concern regarding energy intake in females, as they were also more likely to be low energy reporters. Alternatively, we have previously demonstrated that young, Canadian male athletes are more likely than their female counterparts to use supplements for overall athletic performance [11] and others have found increased use in young males [37].

Our results are limited in that they are based on a single 24-h food recall and there is low reliability for a few dietary supplements, however, these limitations are somewhat offset by the large sample size. Additionally, we did not assess physical stages of maturation and it must be acknowledged that young athletes develop at different rates with considerable variation likely within the groups. The analysis was based on DRI recommendations; however, these are generalized and not specific to the individual or their athletic pursuits. Furthermore, multiple comparisons were made with a 5% level of significance, so it could be expected that up to 5% of results could be due to chance alone. Although caution should be taken when interpreting the findings, the reporting of all potentially important associations was deemed to outweigh the cost of a false positive in this case. Future research should be conducted to either refute or corroborate the findings.

## 5. Conclusions

Athlete support personnel should focus on food sources of vitamin D and calcium, iron, and folate (females). Supplementation is generally unnecessary, with the possible exceptions of vitamin D and iron, under physician supervision. Carbohydrate supplements should be emphasized to balance depletion versus overconsumption. Our research is novel in that, to our knowledge, it provides the first large scale, multi-sport assessment of dietary intakes in pre-adolescent and adolescent Canadian athletes. The information gained can be practically used to inform athlete support personnel regarding common trends and areas of nutritional concern in young athletes.

## Figures and Tables

**Table 1:** Descriptive characteristics.

Descriptive Characteristics	All	Males 11–13 Years	Females 11–13 Years	Males 14–18 Years	Females 14–18 Years
Participants	168	26	37	53	52
Age years	14 (13–16)	12 (11–13)	13(12–13)	15 (15–16)	15 (14.5–17)
Weight kg	56 (12.9)	47 (9.9)	46 (8.7)	67 (12.2)	57 (7.2)
Height m	1.7 (1.6–1.8)	1.6 (1.5–1.6)	1.6 (1.5–1.6)	1.8 (1.8–1.9)	1.7 (1.6–1.7)
BMI kg/m^2^	19.9 (18.3–21.7)	18.5 (16.9–19.9)	18.7 (17.7–20.6)	21.2 (19.6–22.2)	20.5 (19.0–21.9)
Level of Competition					
Club	55	7	14	21	13
Provincial	60	16	19	10	15
National	33	0	3	15	15
International	20	3	1	7	9
Sport Classification					
Endurance	42	5	9	13	15
Intermittent	69	7	16	26	20
Aesthetic	24	1	11	1	11
Power/Strength	33	13	1	13	6
Response Rate					
Total Assigned ID	327				
Food Recall	187 (57%)				
Low Energy Reporters	19 (10%)	2 (7%)	2 (5%)	3 (5%)	12 (19%)
Dietary Supplements	173 (53%)				

Descriptive characteristics are provided for all participants that reported adequate energy intakes on their Food Behaviour Questionnaire. Age, height, and BMI are median (interquartile range) and weight is mean (standard deviation). Values in “[]” under “Response Rate“ refer to the percent of participants who completed the food recall and dietary supplement portions of the questionnaire, as well as those who were removed because they were low energy reporters [17].

**Table 2:** Reported energy and macronutrient intakes.

Nutrient	Male 11–13 (*n* = 26)	Female 11–13 (*n* = 37)	Male 14–18 (*n* = 53)	Female 14–18 (*n* = 52)	*p*
Energy kcal/day	2745 (2165–3555)	2159 (1717–2437)	2905 (2291–3483)	2177 (1764–2540)	
Carbohydrates g/day	386 (282–442)	264 (207–344)	353 (282–464)	281 (218–333)	
Carbohydrates %kcal *	56 (45–61)	54 (49–59)	52 (42–56)	52 (47–59)	0.435
Carbohydrates g/kg BW	8.1 (6.1–10.5)	5.7 (4.5–7.9) ^a^	5.3 (4.3–7.4) ^b^	4.9 (4.4–6.2) ^c^	**<0.001**
Fibre g	25 (20–35)	19 (14–28)	25 (18–33)	23 (19–29)	
Sugar g	144 (115–191)	93 (71–146)	146 (109–183)	106 (81–145)	
Sugar % kcal *	24 (17–28)	20 (16–26)	20 (16–26)	22 (17–25)	0.741
Protein g/day	114 (82–162)	94 (67–112)	123 (104–159)	97 (76–113)	
Protein %kcal	16 (13–20)	16 (15–19)	17 (15–21)	17 (14–20)	0.660
Protein g/kg BW	2.4 (1.6–3.4)	2.0 (1.4–2.6)	2.0 (1.5–2.4) ^b^	1.7 (1.3–2.0) ^c^	**<0.001**
Fat g/day	92 (79–128)	72 (53–95)	108 (88–133)	79 (60–102)	
Fat %kcal *	33 (26–38)	31 (26–36)	33 (28–40)	33 (28–39)	0.700
Fat g/kg BW	2.2 (1.4–2.7)	1.7 (1.2–2.1)	1.7 (1.3–2.1)	1.4 (1.1–1.8) ^c^	**0.002**
Saturated Fat g	33 (27–43)	23 (18–32)	38 (28–47)	24 (17–34)	
Saturated Fat %kcal *	11 (9–13)	10 (8–13)	11 (9–14)	11 (8–14)	0.723
MUFAs g	29 (23–42)	25 (16–33)	35 (29–42)	23 (18–30)	
PUFAs g	13 (9–23)	11 (7–18)	17 (13–24)	12 (9–18)	
Trans Fat g	0.5 (0.1–1.1)	0.2 (0.1–0.9)	0.5 (0.2–0.9)	0.1 (0–0.5)	
Cholesterol g	315 (174–484)	253 (105–388)	408 (271–629)	255 (176–393)	

Intakes are presented as median (interquartile range). BW, body weight; kcal, kilocalories; MUFAs, monounsaturated fatty acids; and PUFA, polyunsaturated fatty acids. Significant differences in %kcal and g/kg/BW were determined by a Kruskal–Wallis non-parametric test and ^a^ difference between genders within age group; ^b^ difference between age group within gender; and ^c^ difference between genders within different age groups were assessed using Dunn’s test for post-hoc pairwise comparisons. Variables with * are normally distributed thus an ANOVA was also performed and the differences remained non-significant.

**Table 3:** Micronutrient intakes from food sources.

Nutrient	Male 11–13 (*n* = 26)	Females 11–13 (*n* = 37)	Males 14–18 (*n* = 53)	Female 14–18 (*n* = 52)	*p*
Thiamin mg/day	1.9 (1.4–2.3)	1.4 (1.0–1.8)	2.1 (1.6–3.0)	1.6 (1.1–2.1)	
%RDA	203 (140–240)	155 (112–204)	175 (137–246)	156 (113–213)	0.073
Riboflavin mg/day	2.9 (2.2–3.9)	2.1 (1.6–3.0)	3.4 (2.6–4.1)	2.1 (1.4–2.7)	
%RDA	317 (248–434)	237 (182–336) ^a^	258 (200–314) ^b^	207 (143–271) ^a,c^	**<0.001**
Niacin mg/day	40 (30–59)	33 (24–43)	45 (37–63)	37 (24–45)	
%RDA	335 (250–494)	274 (200–359)	282 (230–393)	264 (173–325) ^c^	**0.024**
Vitamin B6 mg/day	2.1 (1.4–3.2)	1.3 (1.0–1.9)	2.4 (1.6–3.1)	1.6 (1.2–2.3)	
%RDA	208 (142–315)	133 (104–193) ^a^	185 (127–238)	132 (103–192) ^a,c^	**0.002**
Folate μg/day	412 (314–651)	309 (260–382)	486 (339–642)	357 (244–457)	
%RDA	138 (105–217)	103 (87–127) ^a^	122 (85–161)	89 (61–114) ^a,c^	**<0.001**
Vitamin B12 μg/day	5.5 (3.6–8.4)	4.0 (2.5–5.9)	7.5 (4.2–10.3)	3.5 (2.3–5.6)	
%RDA	303 (198–465)	223 (137–326)	313 (174–429)	147 (95–233) ^a,b,c^	**<0.001**
Vitamin C mg/day	160 (65–262)	97 (54–141)	143 (81–279)	164 (105–240)	
%RDA	356 (143–583)	216 (119–313)	191 (107–372)	252 (161–369)	0.187
Vitamin D μg/day	8.4 (4.7–16.4)	4.3 (1.4–7.4)	9.2 (5.7–15.2)	3.1 (1.7–6.3)	
%RDA	56 (31–109)	29 (10–49) ^a^	62 (38–101) ^c^	21 (11–42) ^a,c^	**<0.001**
Vitamin A RAE μg/day	933 (651–1191)	463 (287–842)	864 (702–1121)	697 (353–1065)	
%RDA	156 (108–199)	77 (48–140) ^a^	96 (78–125) ^b^	100 (50–152) ^c^	**0.005**
Calcium mg/day	1419 (1113–2233)	1040 (715–1492)	1686 (1169–2251)	1090 (625–1377)	
%RDA	109 (86–172)	80 (55–115) ^a^	130 (90–173) ^c^	84 (48–106) ^a,c^	**<0.001**
Iron mg/day	18 (12–20)	13 (10–17)	18 (15–22)	14 (11–17)	
%RDA	228 (150–250)	166 (130–211)	165 (137–195)	91 (72–112) ^a,b,c^	**<0.001**
Zinc mg/day	14 (10–16)	10 (7–13)	15 (12–18)	9 (7–13)	
%RDA	177 (121–204)	121 (88–167) ^a^	138 (107–166)	103 (79–140) ^a,c^	**<0.001**
Potassium mg/day	3934 (3156–5150)	2534 (2125–3716)	4292 (3483–5012)	3240 (2274–3940)	
%AI	87 (70–114)	56 (47–83) ^a^	91 (73–107) ^c^	69 (48–84) ^a,c^	**<0.001**
Sodium mg/day	3476 (2648–4573)	2701 (2089–3388)	3651 (2967–4676)	2928 (2144–3564)	
%AI	232 (177–305)	180 (139–226) ^a^	243 (198–312) ^c^	195 (143–238) ^a^	**<0.001**

Intakes are median (interquartile range). Percent RDA or AI for each athlete was calculated by taking their intake and dividing it by the established RDA or AI for that age and gender [21] and multiplying by 100. Significant differences in percent RDA or AI were determined by a Kruskal–Wallis non-parametric test and ^a^ difference between genders within age group; ^b^ difference between age group within gender; and ^c^ difference between genders within different age groups were assessed using Dunn’s test for post-hoc pairwise comparisons.

**Table 4:** Canada’s Food Guide servings.

Nutrient	Male 11–13 (*n* = 26)	Female 11–13 (*n* = 37)	Male 14–18 (*n* = 53)	Female 14–18 (*n* = 52)	*p*
Vegetables and Fruits	6.5 (4.0–10.0)	4.0 (3.0–7.0)	7.0 (4.0–9.0)	7.0 (5.0–9.0)	
Met %	62 (*n* = 16)	35 (*n* = 13)	40 (*n* = 21)	54 (*n* = 28)	0.093
Grains	9.0 (6.0–11.0)	6.0 (4.0–9.0)	8.0 (6.0–11.0)	7.0 (5.0–8.0)	
Met %	81 (*n* = 21)	65 (*n* = 24)	68 (*n* = 36)	65 (*n* = 34)	0.516
Milk and Alt.	3.0 (3.0–6.0)	3.0 (2.0–4.0)	4.0 (3.0–6.0)	2.0 (1.0–3.5)	
Met %	77 (*n* = 20)	57 (*n* = 21)	76 (*n* = 40)	48 (*n* = 25)	**0.010**
Meat and Alt.	3.0 (1.0–3.0)	3.0 (2.0–3.0)	3.0 (2.0–5.0)	3.0 (2.0–3.0)	
Met %	89 (*n* = 23)	89 (*n* = 33)	59 (*n* = 31)	77 (*n* = 40)	**0.002**
Other	4.0 (2.0–6.0)	3.0 (2.0–6.0)	4.0 (3.0–6.0)	3.5 (2.0–6.5)	
Met %	N/A	N/A	N/A	N/A	

Eating Well with Canada’s Food Guide [22] servings are presented as median (interquartile range). Percent met was calculated as the percent of athletes meeting the minimum recommended daily amounts. Significant differences in the percent of athletes in the groups meeting the minimum recommendations were determined by a Pearson’s Chi-squared test.

**Table 5:** Dietary supplements and ergogenic aids.

Supplement % Athletes	Males 11–13 (*n* = 26)			Females 11–13 (*n* = 36)			Males 14–18 (*n* = 52)			Females 14–18 (*n* = 59)			
	R	O	N	R	O	N	R	O	N	R	O	N	*p*
MVMM	46	15	39	36	19	44	40	19	40	22	27	51	0.346
B Vitamins	0	8	92	6	6	89	6	17	77	3	10	86	0.482
Vitamin C	15	23	62	19	31	50	27	23	50	19	27	54	0.862
Vitamin E	0	4	96	3	11	86	2	6	92	3	12	85	0.747
Vitamin D *	19	0	81	19	3	78	14	0	87	24	0	76	0.453
Vitamin Water	8	39	54	3	61	36	10	46	44	7	48	46	0.629
Iron	0	0	100	3	3	94	2	6	92	5	12	83	0.251
Calcium	0	0	100	6	8	86	8	10	83	3	7	90	0.471
Magnesium	0	4	96	3	3	94	2	6	92	0	2	98	0.735
Protein Powder	12	19	69	8	25	67	25	42	33	12	48	41	**0.004**
Beta Alanine	4	0	96	0	0	100	0	6	94	0	0	100	**0.047**
BCAA	4	0	96	3	0	97	2	6	92	3	2	95	0.598
Glutamine	4	0	96	0	0	100	0	4	96	0	2	98	0.230
Glucosamine	0	0	100	0	0	100	2	4	94	0	5	95	0.495
Fatty Acids	19	8	73	17	11	72	15	4	81	3	17	80	0.090
Sport Drink	15	65	19	8	69	22	19	71	10	7	70	24	0.264
Recovery Drink	15	8	77	0	14	86	4	23	73	5	17	78	0.109
Energy Drink	0	4	96	0	3	97	0	33	67	0	22	78	**0.001**
Sport Bar	15	46	39	14	69	17	33	50	17	25	51	24	0.110
Creatine	4	0	96	0	0	100	2	8	90	2	0	98	0.092
Caffeine	0	0	100	0	0	100	0	2	98	0	0	100	0.505
Gel/Gummy	8	46	46	0	47	53	2	29	69	0	37	63	0.079
Plant Extracts	4	31	65	3	39	58	2	31	67	3	37	59	0.968
Probiotics	4	19	77	3	19	78	0	14	87	0	20	80	0.559

Dietary supplement use is presented as percent of athletes who completed the dietary supplement portion of the questionnaire (*n* = 173). * Vitamin D was determined from responses to “Other Vitamins”. MVMM, multivitamin-multimineral; BCAA, branched chain amino acids; R, regularly; O, occasionally; and N, never. Significant differences in the percent of athletes in the groups consuming the supplement were determined by a Pearson’s Chi-squared test.
